# Differential impact of biologically effective dose in distal versus proximal gamma knife targets for trigeminal neuralgia

**DOI:** 10.3389/fneur.2025.1614981

**Published:** 2025-08-25

**Authors:** Hao Deng, Yuan Gao, Yang Wu, Mengqi Wang, Linglong Xiao, Runlin Chen, Zhujun Zhang, Wei Pan, Wei Wang

**Affiliations:** ^1^Department of Neurosurgery, West China Hospital, Chengdu, China; ^2^Gamma Knife Center, West China Hospital, Chengdu, China

**Keywords:** biologically effective dose, gamma knife radiosurgery, primary trigeminal neuralgia, pain relief, recurrence

## Abstract

We report the results of a long-term follow-up series in our center to verify the impact of biologically effective dose (BED) on the efficacy and safety of Gamma Knife radiosurgery (GKS) in the treatment of primary trigeminal neuralgia (TN). A total of 138 consecutive cases of primary TN receiving GKS were included. A 4-mm collimator was used for all cases, and a median central dose of 85 Gy (range 70–90 Gy) was prescribed. The Barrow Neurological Institute Pain Scale was adopted to evaluate the severity of TN. The median follow-up period was 65.5 months (range 12–147 months). Overall, 123 (89.1%) patients eventually achieved effective relief. The influence of BED on treatment outcomes varied by target location. For patients with distal targets, BED was a significant predictor of treatment failure (OR: 0.996, 95% CI: 0.992–0.999, *p* = 0.02) and post-GKS complications (OR: 1.002, 95% CI: 1.000–1.004, *p* = 0.01). However, BED did not significantly influence outcomes in the proximal target subgroup, either for treatment failure or complications. No significant association was found between BED and long-term outcomes in the entire cohort or in any subgroup analysis. Adjusting GKS doses according to BED for the distal target may optimize clinical outcomes in TN patients.

## 1 Introduction

Trigeminal neuralgia (TN) is chronic neuropathic pain with a high rate of recurrence. The incidence of TN is approximately 0.03%, with a male-to-female ratio of approximately 33% (1). According to the latest classification, TN is classified into classical, secondary, and idiopathic (2). Classical TN refers to TN caused by microvascular compression, which is the most common type. Recurrent facial pain has a serious impact on a patient’s quality of life and even leads to disabilities of social function (3–6). The anticonvulsant agents carbamazepine and oxcarbazepine are still the first-choice treatment for the management of TN, regardless of the type (7). For patients with poor drug response or severe side effects, surgical intervention is necessary. Gamma knife radiosurgery (GKS) is a well-established procedure for TN, and many studies have verified its efficacy and safety (8–10).

The biologically effective dose (BED) is a parameter used to quantify the biological effectiveness of any radiotherapy treatment (11). It accounts for cellular deoxyribonucleic acid repair during radiation exposure (12). The effect of BED on GKS has been investigated in several diseases, including pituitary adenoma, arteriovenous malformation and meningioma (13–17). Recently, a multi-center study reported the relevance of BED and clinical outcomes in TN patients receiving GKS, which attracted more attention to the application of BED in GKS (18). An anatomical feature of particular relevance is the myelin transition zone within the trigeminal nerve, where central myelin produced by oligodendrocytes transitions to peripheral myelin produced by Schwann cells (19, 20). Given the histological heterogeneity along the trigeminal nerve, it is plausible that different target locations may exhibit varying sensitivity to radiation. Consequently, BED may produce differential therapeutic effects depending on the irradiated target. Further verification of the role of BED in GKS may be helpful to optimize the treatment plan of GKS in the future. Therefore, we report the results of a long-term follow-up series in our center to further verify the impact of BED on the efficacy and safety of GKS when different targets were used in the treatment of primary TN.

## 2 Materials and methods

### 2.1 Study design and patient selection

This study was designed as an observational, noncomparative, retrospective study and follows the principles outlined in the Declaration of Helsinki. The strengthening the reporting of observational studies in epidemiology (STROBE) statement was implemented to report this study (21). This study obtained approval from the Institutional Review Board of West China Hospital, Sichuan University (2023/1534). Due to the retrospective nature of the study, the Institutional Review Board of West China Hospital, Sichuan University waived the need of obtaining informed consent. All methods were performed in accordance with the relevant guidelines and regulations. We retrospectively reviewed the records of patients presenting with medically refractory TN treated with GKS between January 2011 and December 2021 at the Gamma Knife Center, West China Hospital (Chengdu, China). The inclusion criteria were at least 1 year of follow-up, refusal to other surgical interventions, and primary TN (*n* = 213). Patients receiving multiple shots in a single treatment were excluded to reduce confounding factors (*n* = 56). Patients previously treated with GKS were also excluded (*n* = 8). Eleven patients were excluded for inadequate follow-up. Finally, a total of 138 TN patients were included. All patients included fulfilled the diagnostic criteria for TN established by the Headache Classification Committee of the International Headache Society (IHS) (22). Types of TN was divided into TN1 (typical TN without background pain) and TN2 (atypical TN with background pain).

### 2.2 Baseline data and follow-up

We consulted the electronic medical records of all included patients to obtain demographic data and disease conditions. GKS parameters were collected by consulting the GKS records sheet on the GammaPlan 11.0 system.^1^ Follow-up occurred at 3 months, 6 months, and 1 year after GKS. The first three follow-up visits were conducted on an outpatient basis so that the patients’ condition could be evaluated accurately and necessary adjustments of medication could be made. For patients with long-term stable disease, follow-up was conducted at least once a year and could be in the form of telephone or online interviews.

### 2.3 GKS procedure

From January 2011 to December 2019, the Leksell C system (Elekta Instrument, Stockholm, Sweden) was used for 111 patients. Then, the other 27 received GKS performed using the ICON system (Elekta Instrument, Stockholm, Sweden). A Leksell stereotactic frame (Elekta Instruments, Stockholm, Sweden) was placed for patients under local anesthetic. One-mm T2-weighted constructive interference in steady-state sequences or 1-mm T1-weighted sequences without contrast was used for preoperative MRI location. The proximal target was defined as an isocenter positioned on the proximal segment of the trigeminal nerve, with the 50% isodose line encompassing the emergence of the trigeminal nerve. In contrast, the distal target referred to the segment of the trigeminal nerve located distally in anterior pontine cistern. Due to the lack of conclusive evidence favoring one target over the other, no standardized protocol for target selection has been established in our center. Decisions are primarily guided by the presence of preexisting facial numbness, anatomical clarity on imaging, and surgeon preference. The GammaPlan software (Elekta Instruments) was used by senior neurosurgeons to identify anatomical structures and make a treatment plan. A 4-mm collimator was used for all cases. The V_50_ was contoured retrospectively using the GammaPlan, and ID_50_ were calculated using dose-volume histograms. We adopted the model described by Jones et al. in 2019 to calculate BED (22). An *α*/*β* ratio of 2.47 Gy was used. The model based on biexponential DNA repair kinetics compensated for the impact of treatment duration on radiation biological effects. The detailed BED calculation was presented in Supplementary material.

### 2.4 Outcome measures

All cases were evaluated by the Barrow Neurological Institute Pain Scale (BNI-PS): BNI-PS I, no pain and not requiring drugs; BNI-PS II, occasional pain and not requiring drugs; BNI-PS IIIa, no pain but requiring continuous drugs; BNI-PS IIIb, mild pain and controlled with drugs; BNI-PS IV, moderate pain and not adequately controlled with drugs; and BNI-PS V, severe pain and no relief with drugs (23). The patients included were all at the level of BNI-PS IV-V before GKS. Effective pain relief was defined as BNI-PS grades decreasing to I-IIIb after GKS. Complete relief means that patients no longer need to take drugs for TN. Failure refers to BNI-PS grades remaining IV-V after GKS. Recurrence was defined as BNI-PS change from I-IIIb to IV-V in those with effective relief. Complications were determined by both physical examination and self-reports of patients. Post-GKS sensory dysfunction was evaluated using BNI Facial Hypesthesia Scale (23). Corneal anesthesia was identified based on corneal reflex testing. Masticatory weakness and changes in salivation were recorded based on patient-reported symptoms. The GKS-related complications were evaluated according to the following criteria: I, no GKS-related complication; II, mild complications having no impact on daily life; III, complications having some effect on daily life; and IV, complications having a severe effect on daily life.

### 2.5 Statistical analyses

Statistical analyses and plot making were performed with R 4.1.3 software^2^ and the packages including survival, survminer, glmnet, and pROC. The time-to-event data were estimated using the Kaplan–Meier method stratified according to variables and compared using a two-sided log-rank test. Cox regression analysis was performed to explore the predictors of long-term pain relief. The hazard ratio (HR) with a 95% confidence interval (CI) was presented to reflect the association between variables and long-term outcomes. The optimal cutoff value of continuous variables in survival analyses was determined using the maximally selected rank statistics from the ‘maxstat’ R package. For dichotomous outcomes, we used logistic regression to explore relevant influencing factors, and the odds ratio (OR) with 95% CI was used to present the results. We used the ROC to determine the optimal cutoff value of continuous variables in logistic regression. The cutoff that maximized the Youden index (sensitivity + specificity − 1) was selected as the optimal threshold for dichotomizing continuous variables. Variables with *p <* 0.1 in univariate analysis were included in multivariate analysis. Variables that were colinear or derived from one another were not included together in the multivariate analysis model. The AUC was calculated to compare the predictive strength of collinear variables. A *p* value of <0.05 was considered statistically significant.

## 3 Results

### 3.1 Patient characteristics and GKS parameters

The median age of patients at the time of GKS was 65 years (range 44–90). Females accounted for 63.0%. Only 1 patient had bilateral TN. There were 79 patients (57.2%) with pain on the right side and 59 patients (42.8%) had pain on the left side. The most frequently affected branch of the trigeminal nerve was V2 (41.3%). The median duration between pain onset and GKS was 60 months (range 1–360 months). Microvascular compression (MVC) existed in 42 cases (30.4%) on the side being irradiated. Twenty-five patients (18.1%) had previous surgical treatment. Three patients received 2 kinds of surgeries. The baseline characteristics of the included patients are listed in Table 1. The proximal target was used for 62 patients, and the distal target was used for the others. The proximal targeting group received significantly lower physical dose and BED (*p* < 0.05), while the distal targeting group exhibited a significantly lower mean CDR (*p* < 0.05).

**Table 1:** Characteristics of included patients.

Variables	Entire cohort	Proximal target	Distal target	*p* ^a^
Sex, *n* (%)				0.835
Male	51 (37.0)	24 (38.7)	27 (35.5)	
Female	87 (63.0)	38 (61.3)	49 (64.5)	
Age at GKS, years	63.69 ± 10.15	66 ± 9.68	61.8 ± 10.2	0.015
Duration between TN onset and GKS, months	77.3 ± 70.19	93.32 ± 83.41	64.22 ± 54.36	0.015
Type of TN, *n* (%)				0.999
TN1	125 (90.6)	56 (90.3)	69 (90.8)	
TN2	13 (9.4)	6 (9.7)	7 (9.2)	
Distribution of pain, *n* (%)				
V1	6 (4.3)	3 (4.8)	3 (3.9)	0.846
V2	57 (41.3)	27 (43.5)	30 (39.4)	
V3	18 (13.0)	8 (12.9)	10 (13.2)	
V1 + V2	6 (4.3)	2 (3.2)	4 (5.3)	
V2 + V3	41 (29.7)	16 (25.8)	25 (32.9)	
V1 + V2 + V3	10 (7.2)	6 (9.7)	4 (5.3)	
Previous intervention, *n* (%)				0.065
No previous surgery	113 (81.9)	46 (74.2)	67 (88.2)	
MVD	8 (5.8)	6 (9.7)	2 (2.6)	
Radiofrequency thermocoagulation	11 (8.0)	6 (9.7)	5 (6.6)	
Balloon compression	3 (2.2)	3 (4.8)	0 (0)	
Glycerol/ethanol injection	6 (4.3)	4 (6.5)	2 (2.6)	
Central dose, Gy	83.14 ± 5.64	79.95 ± 5.74	85.75 ± 4	<0.001
CDR, Gy/min	2.11 ± 0.61	2.3 ± 0.58	1.96 ± 0.59	<0.001
BED, Gy_2.47_	1968.17 ± 272.88	1866.47 ± 239.18	2051.14 ± 271.97	<0.001

TN, trigeminal neuralgia; GKS, Gamma knife radiosurgery; MVD, microvascular decompression; CDR, dose rate of Co^60^.

Continuous variables were presented as mean ± se.

a *p*-value for categorical variables was calculated by Chi-square test, for continuous variables was calculated by single factor ANOVA.

### 3.2 Initial pain relief

Overall, 123 (89.1%) patients eventually achieved effective relief with a median latency of 2 months. GKS was considered to have failed in 15 patients (10.9%). Complete pain relief (The Barrow Neurological Institute Pain Scale, BNI-PS I-II) was observed in 67.4% of patients. Outcomes and complications are shown in Table 2. BED <1975 Gy_2.47_ (OR: 4.07, 95%: 1.22–18.45, *p =* 0.04) was the only predictor of failure after GKS for the entire cohort. TN2 (OR: 0.18, 95%CI: 0.05–0.59, *p =* 0.01) and a history of previous surgeries (OR: 0.36, 95%CI: 0.15–0.88, *p =* 0.03) were negative factors for complete pain relief. Physical dose (*p* = 0.43) and CDR (*p =* 0.38) were not significant factors for initial pain relief. In the subgroup analysis, BED was found to be predictive of failure for the distal target. As a continuous variable, BED had an OR of 0.996 (95%CI: 0.992–0.999, *p =* 0.02). When categorized as a binary variable (with a threshold of BED <1850 Gy_2.47_), the OR was 19.44 (95%CI: 3.16–376.92, *p =* 0.01). Additionally, the integral dose (mean dose × tissue volume) inside the 50% isodose line (ID_50_) was significantly associated with failure for the proximal target (OR: 14.56, 95%CI: 1.99–192.39, *p =* 0.02), as was the nerve volume inside the 50% isodose line (V_50_) (OR: 4.85, 95%CI: 1.41–22.49, *p =* 0.02). Increased ID_50_ and V_50_ corresponded to increased risk of failure. Univariate analysis results for failure are summarized in Table 3, with the multivariate analysis results provided in Supplementary Table S1.

**Table 2:** Initial pain relief and adverse events.

Variables	Entire cohort	Proximal target	Distal target	*p* ^a^
Effective pain relief, *n* (%)	123 (89.1)	55 (88.7)	68 (89.5)	0.999
Complete pain relief, *n* (%)	93 (67.4)	41 (66.1)	52 (68.4)	0.918
Failure, *n* (%)	15 (10.9)	7 (11.3)	8 (10.5)	0.999
Recurrence at last FU, *n* (%)	26 (18.9)	12 (19.4)	14 (18.4)	0.999
BNI-PS at last FU, *n* (%)				0.165
I	90 (65.2)	38 (61.3)	52 (68.4)	
II	3 (2.2)	3 (4.8)	0 (0)	
IIIa	15 (10.9)	6 (9.7)	9 (11.8)	
IIIb	15 (10.9)	8 (12.9)	7 (9.2)	
IV	12 (8.7)	7 (11.3)	5 (6.6)	
V	3 (2.2)	0 (0)	3 (3.9)	
Presence of complications, *n* (%)	61 (44.2)	24	37	0.106
Complications, *n* (%)				0.111
Hypoesthesia	52 (37.7)	17 (27.4)	35 (46.0)	
Paresthesia	2 (1.4)	2 (3.2)	0 (0)	
Salivation	19 (13.8)	9 (14.5)	10 (13.2)	
Corneal anesthesia	6 (4.3)	4 (6.5)	2 (2.6)	
Masticatory weakness	2 (1.4)	1 (1.6)	1 (1.3)	
Oral mucosal hypoesthesia	4 (2.8)	3 (4.8)	1 (1.3)	
Severity of complications, *n* (%)				0.478
I	77 (55.8)	37 (59.7)	40 (52.6)	
II	54 (39.1)	21 (33.9)	33 (43.4)	
III	7 (5.1)	4 (6.4)	3 (3.9)	
IV	0 (0)	0 (0)	0 (0)	

FU, follow-up; BNI-PS, Barrow Neurological Institute Pain Scale.

Continuous variables were presented as mean ± se.

a *p*-Value for categorical variables was calculated by Chi-square test, for continuous variables was calculated by single factor ANOVA.

**Table 3:** Univariate logistic regression for failure.

Variables	Entire cohort		Proximal target		Distal target	
	OR (95%CI)	*p*	OR (95%CI)	*p*	OR (95% CI)	*p*
Age	1.02 (0.97–1.08)	0.40	0.94 (0.86–1.02)	0.16	1.00 (0.93–1.08)	0.95
Sex	0.59 (0.16–1.83)	0.58	NA	0.99	0.51 (0.11–2.34)	0.37
Duration between onset and GKS	0.99 (0.99–1.00)	0.32	1.00 (0.99–1.01)	0.94	1.00 (0.99–1.02)	0.08
Type of TN	0.35 (0.09–1.73)	0.15	1.67 (0.08–12.96)	0.66	4.20 (0.53–24.9)	0.13
BNI-PS before GKS	0.70 (0.22–2.70)	0.57	NA	0.99	3.25 (0.7–15.22)	0.12
Previous surgery	0.39 (0.12–1.36)	0.12	2.42 (0.43–12.42)	0.28	2.90 (0.38–15.79)	0.24
Presence of MVC	1.23 (0.39–4.66)	0.74	0.65 (0.09–3.31)	0.62	1.00 (0.14–4.83)	1.00
Central dose	1.04 (0.94–1.14)	0.43	1.06 (0.80–1.40)	0.66	0.70 (0.46–1.00)	0.06
CDR	1.51 (0.62–3.98)	0.38	1.70 (0.42–8.27)	0.47	0.16 (0.01–0.85)	0.08
V_50_	0.75 (0.42–1.39)	0.34	4.85 (1.41–22.49)	0.02*	0.78 (0.3–1.72)	0.58
ID_50_	0.68 (0.29–1.68)	0.38	14.56 (1.99–192.39)	0.02*	0.66 (0.16-2.11)	0.52
BED	1.002 (0.999–1.003)	0.16	1.001 (0.998–1.004)	0.42	0.996 (0.992–0.999)	0.02*
BED <1975 Gy_2.47_ (entire cohort)	4.07 (1.22–18.47)	0.04*	4.05 (0.79–30.07)	0.113	19.44 (3.16–376.92)	0.01*
BED <1829 Gy_2.47_ (proximal)						
BED <1850 Gy_2.47_ (distal)						
Target	0.92 (0.31–2.78)	0.89	–	–	–	–

OR, odds ratio; CI, confident interval; GKS, Gamma knife radiosurgery; TN, trigeminal neuralgia; BNI-PS, Barrow Neurological Institute Pain Scale; MVC, microvascular decompression; CDR, dose rate of Co^60^; V_50_, nerve volume inside the 50% isodose line; ID_50_, integral dose inside the 50% isodose line; BED, biologically effective dose.

*p < 0.05.

### 3.3 Long-term outcomes

**Figure 1:**
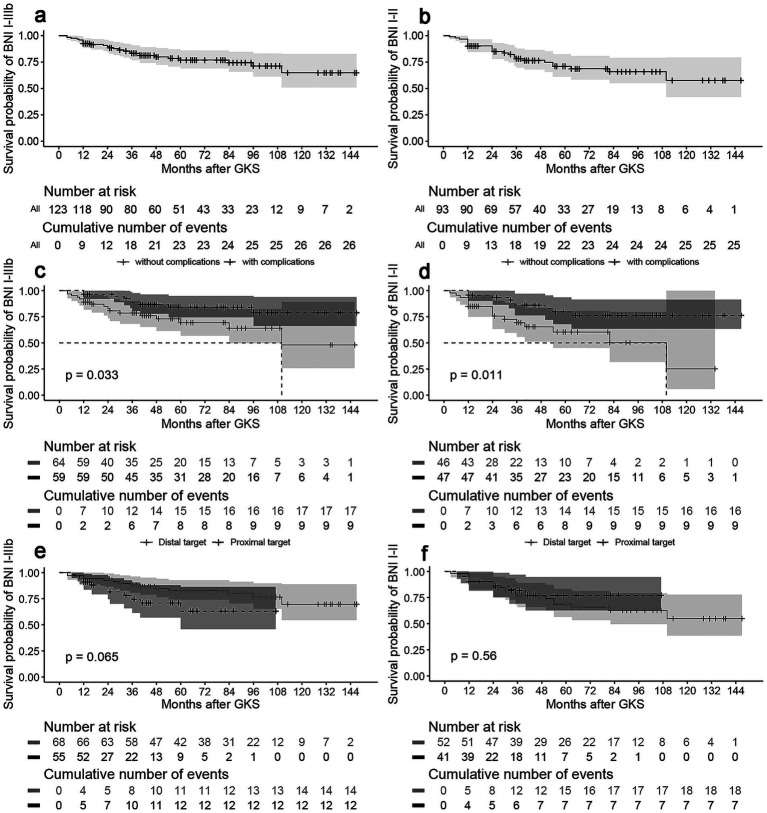
**(a)** Actuarial probability of maintaining effective pain relief (BNI-PS I-IIIb). The probabilities of maintaining effective pain relief at 1, 3, 5, 7, and 10 years after GKS were 92.7, 83.5, 77.0, 69.9, and 64.9%. **(b)** Actuarial probability of maintaining complete pain relief (BNI-PS I-II). At 1, 3, 5, 7, and 10 years after GKS, the probabilities of maintaining complete relief were 90.3, 78.2, 70.9, 65.9, and 57.6%. **(c)** Survival curves for effective pain relief grouped by the appearance of complications. Patients with complications was at a lower risk of recurrence (*p* < 0.05). **(d)** Survival curves for complete pain relief grouped by the appearance of complications. Patients with complications was more likely to maintain pain relief without medication (*p* < 0.05). **(e)** Survival curves for effective pain relief grouped by target. Patients treated with different targets showed no significant difference in recurrence (*p* > 0.05). **(f)** Survival curves for complete pain relief grouped by target. Patients treated with different targets showed no significant difference in maintaining complete relief (*p* > 0.05).

The median follow-up time was 65.5 months (range 12–147 months). During follow-up, recurrence occurred in 26 (18.9%) patients, and 4 patients died of other diseases. The probabilities of maintaining effective pain relief (BNI-PS I-IIIb) at 1, 3, 5, 7, and 10 years after GKS were 92.7, 83.5, 77.0, 69.9, and 64.9%, respectively (Figure 1a). In the patients with recurrence, 13 received second-time GKS, and 13 received other surgical procedures. Twenty patients achieved effective relief after the additional procedure. V_50_, ID_50_ and the presence of GKS-related complications were significant factors for recurrence. The risk of recurrence in patients with complications dropped by approximately 60% (HR: 0.41, 95%CI: 0.18–0.93, *p =* 0.04) (Figure 1c). Increased V_50_ (HR: 0.44, 95%CI: 0.26–0.75, *p =* 0.002) and ID_50_ (HR: 0.32, 95%CI: 0.15–0.71, *p =* 0.004) reduced the risk of recurrence. In subgroup analyses, TN2 (HR: 4.54, 95% CI: 1.20–17.18, *p =* 0.03) and the presence of complications (HR: 0.25, 95% CI: 0.06–0.93, *p =* 0.04) were predictive factors for recurrence in the proximal subgroup. For the distal target, V_50_ (HR: 0.35, 95%CI: 0.17–0.71, *p =* 0.004) and ID_50_ (HR: 0.24, 95% CI: 0.08–0.67, *p =* 0.01) were significantly associated with recurrence. Furthermore, patients receiving ID_50_ > 2 mJ had an 89% reduced risk of recurrence (HR: 0.11, 95%CI: 0.01–0.87, *p =* 0.04). BED did not significantly influence recurrence risk in either the entire cohort or any of the subgroups. The results of the univariate analysis for recurrence are presented in Table 4, while the multivariate analysis results can be found in Supplementary Tables S2, S3. At 1, 3, 5, 7, and 10 years after GKS, the probabilities of maintaining complete relief (BNI-PS I-II) were 90.3, 78.2, 70.9, 65.9, and 57.6%, respectively (Figure 1b). The presence of GKS-related complications was the only significant predictor for maintenance of complete relief (HR: 0.36, 95%CI: 0.17–0.82, *p =* 0.02) (Figure 1d). The target locations had no significant impact on long-term outcomes (Figures 1e,f).

**Table 4:** Univariate COX regression for recurrence.

Variables	Entire cohort		Proximal target		Distal target	
	HR (95%CI)	*p*	HR (95%CI)	*p*	HR (95%CI)	*p*
Age	0.99(0.96–1.03)	0.77	0.98 (0.92–1.04)	0.49	0.99 (0.94–1.04)	0.72
Sex	0.57 (0.26–1.23)	0.15	0.39 (0.12–1.29)	0.13	0.91 (0.30–2.71)	0.86
Duration between onset and GKS	1.00 (0.99–1.01)	0.23	0.99 (0.98–1.00)	0.17	1.00 (0.99–1.01)	0.57
Type of TN	2.10 (0.72–6.13)	0.17	4.54 (1.20–17.18)	0.03*	0.83 (0.11-6.41)	0.86
BNI-PS before GKS	0.40 (0.12–1.35)	0.14	1.02 (0.23–4.73)	0.97	0.20 (0.03–1.52)	0.12
Previous surgery	1.13 (0.39–3.27)	0.82	2.13 (0.62–7.30)	0.23	NA	0.99
Presence of MVC	1.63 (0.73–3.61)	0.23	1.63 (0.52–5.05)	0.40	1.34 (0.42–4.30)	0.62
Central dose	0.99 (0.91–1.07)	0.81	1.02 (0.82–1.27)	0.84	1.07 (0.79–1.44)	0.68
CDR	1.06 (0.56–2.02)	0.86	0.68 (0.23–2.04)	0.50	1.46 (0.61–3.50)	0.40
V_50_	0.44 (0.26–0.75)	0.002*	0.70 (0.29-1.73)	0.44	0.35 (0.17–0.71)	0.004*
ID_50_	0.32 (0.15–0.71)	0.004*	0.64 (0.17-2.39)	0.51	0.24 (0.08–0.67)	0.01*
ID_50_ > 1.9 mJ (entire cohort and proximal target)	0.37 (0.14–0.99)	0.04*	0.65 (0.14-3.04)	0.6	0.11 (0.01–0.87)	0.04*
ID_50_ > 2 mJ (distal target)						
BED	1.00 (0.999–1.002)	0.58	1.001 (0.999–1.003)	0.55	1.001 (0.999–1.004)	0.29
BED>1853Gy_2.47_ (entire cohort)	1.80 (0.71–4.57)	0.22	1.63 (0.42–6.25)	0.48	5.12 (0.66–39.67)	0.12
BED>2237Gy_2.47_ (proximal)						
BED>1955Gy_2.47_ (distal)						
Target	2.13 (0.94–4.84)	0.07	–	–	–	–
Presence of complications	0.43 (0.19–0.96)	0.04*	0.25 (0.06–0.93)	0.04*	0.59 (0.20–1.72)	0.34

HR, hazard ratio; CI, confident interval; GKS, Gamma knife radiosurgery; TN, trigeminal neuralgia; BNI-PS, Barrow Neurological Institute Pain Scale; MVC, microvascular decompression; CDR, dose rate of Co^60^; V_50_, nerve volume inside the 50% isodose line; ID_50_, integral dose inside the 50% isodose line; BED, biologically effective dose.

*p < 0.05.

### 3.4 GKS-related complications

GKS-related complications occurred in 61 patients, with an incidence of 44.2% (Table 2). The most common complication was facial sensation dysfunction, including hypoesthesia and paresthesia (39.1%). Complications in most patients (39.1%) were mild, with a grade of II. No patient reported severe complications (IV). Nineteen (13.8%) patients experienced at least two types of complications. Univariate analysis demonstrated that both central dose (OR: 1.40, 95%CI: 1.10–1.83, *p =* 0.01) and BED (OR: 1.002, 95%CI: 1.000–1.004, *p =* 0.01) were significant predictors of complications across the entire cohort. However, subgroup analysis found these factors were significant only in the distal subgroup (*p =* 0.01). For the distal target, the AUC for BED and complications was 0.69, slightly higher than that for the central dose (AUC = 0.66) (Supplementary Figure 1). Sex was associated with complications for the distal target. The risk of post-GKS complications in females was approximately three times higher than in males (HR: 2.97, 95%CI: 1.07–8.73, *p =* 0.04). The results of the univariate analysis for post-GKS complications are presented in Table 5, while the multivariate analysis results are available in Supplementary Tables S4, S5.

**Table 5:** Univariate logistic regression for post-GKS complication.

Variables	Entire cohort		Proximal target		Distal target	
	OR (95%CI)	*p*	OR (95%CI)	*p*	OR (95%CI)	*p*
Age	1.00 (0.96–1.03)	0.83	1.00 (0.95–1.06)	1.00	1.00 (0.96–1.05)	0.96
Sex	2.05 (1.00–4.26)	0.05	1.09 (0.38–3.18)	0.88	3.44 (1.3–9.81)	0.02*
Duration between onset and GKS	1.00 (0.99–1.01)	0.10	1.01 (1.00–1.01)	0.09	1.00 (1.00–1.01)	0.36
Type of TN	0.35 (0.08–1.20)	0.12	0.29 (0.01–1.94)	0.27	0.39 (0.05–1.94)	0.28
BNI-PS before GKS	2.09 (0.92–4.92)	0.08	2.24 (0.53–10.01)	0.27	1.86 (0.67–5.42)	0.24
Previous surgery	0.64 (0.81–1.94)	0.64	0.65 (0.18–2.09)	0.48	1.37 (0.33–5.95)	0.66
Presence of MVC	0.80 (0.38–1.67)	0.56	0.77 (0.26–2.21)	0.63	0.93 (0.32–2.65)	0.89
Central dose	1.10 (1.03–1.17)	0.01*	1.12 (0.93-1.35)	0.23	1.40 (1.10–1.83)	0.01*
Central dose >84.5 Gy (entire cohort)	3.21 (1.61–6.59)	0.001*	2.47 (0.87–7.42)	0.10	5.16 (1.64–19.88)	0.01*
Central dose >78 Gy (proximal)						
Central dose >82 Gy (distal)						
CDR	1.12 (0.64-1.95)	0.69	0.7 (0.28–1.70)	0.43	1.95 (0.90–4.39)	0.10
V_50_	0.89 (0.59–1.33)	0.57	0.86 (0.41–1.78)	0.68	0.87 (0.52–1.42)	0.57
ID_50_	1.05 (0.58–1.88)	0.88	0.95 (0.31–2.85)	0.92	0.99 (0.48–2.03)	0.97
BED	1.002 (1.000–1.003)	0.01*	0.998 (1.003-1.001)	0.62	1.002 (1.000–1.004)	0.01*
BED >2086Gy_2.47_ (entire cohort)	2.94 (1.45–6.05)	0.003*	0.64 (0.22–1.79)	0.40	3.29 (1.23–9.44)	0.02*
BED >1822Gy_2.47_ (proximal)						
BED >2245Gy_2.47_ (distal)						
Target	0.67 (0.33–1.31)	0.24	–	–	–	–

OR, odds ratio; CI, confident interval; GKS, Gamma knife radiosurgery; TN, trigeminal neuralgia; BNI-PS, Barrow Neurological Institute Pain Scale; MVC, microvascular decompression; CDR, dose rate of Co^60^; V_50_, nerve volume inside the 50% isodose line; ID_50_, integral dose inside the 50% isodose line; BED, biologically effective dose.

*p < 0.05.

## 4 Discussion

In this retrospective study, we focused on the impact of BED on the safety and efficacy of GKS in primary TN. The influence of BED on treatment outcomes varied by target location. BED was a significant predictor for treatment failure and post-GKS complications in the distal target subgroup. However, BED did not significantly influence outcomes in the proximal target subgroup, either for treatment failure or complications. No significant association was found between BED and long-term outcomes in the entire cohort or any of the targeting subgroups.

The concept of BED was first proposed in 1989 (11). Although it has been applied in general radiotherapy for many years, a model suitable for BED calculations in GKS was not proposed until recent years (24). Before that, people tried to evaluate the impact of CDR and physical dose on GKS. Two early studies did not find a significant difference in pain control or complications for TN patients receiving GKS delivered by varying CDR (25, 26). However, Lee et al. (27) in 2015 and Barzaghi et al. (28) in 2021 reported heterogeneous results showing that CDR > 2.0 Gy/min or 2.5 Gy/min was associated with a lower likelihood of recurrence (*p <* 0.05). In our study, CDR < 1.466 Gy/min might be a predictor for failure after GKS in the overall group, but the difference was not statistically significant (HR: 3.06, 95%CI: 0.94–9.41, *p =* 0.05). Current evidence does not conclude an optimal physical dose for TN. The most commonly used central dose is 70–90 Gy (8, 29, 30). 90 Gy is now viewed as an appropriate upper limit because a central dose larger than 90 Gy might sharply increase the risk of GKS-related complications (10). Varying CDR make a difference in the delivery time of a given physical dose. As a result, the same prescribed dose produced different BED. However, people were usually not aware of the need to compensate for variations in biological effectiveness caused by varying delivery time when making GKS plans. The effect of BED on GKS has been verified in several diseases. BED>45 Gy_2.47_ was a significant predictive factor for new hypopituitarism after GKS in patients with pituitary adenoma, and high BED levels might be associated with better endocrine remission in patients with acromegaly and Cushing’s disease (13, 14, 31). For patients with meningiomas treated with GKS, BED>50 Gy_2.47_ was associated with a lower incidence of local recurrence (*p =* 0.03) (15). BED was a significant predictor of obliteration of unruptured arterial venous malformations (AVMs) after upfront GKS (16, 17). According to our study, the physical dose did not impact initial pain relief, whether analyzed as a continuous or dichotomous variable. A recent multicenter study by Warnick et al. (18) suggested that BED ≥2,100 Gy_2.47_ was a significant predictor of initial pain relief for the distal target (HR: 1.46, 95%CI: 1.05–2.03, *p* = 0.03) and physical dose ≥85Gy was a significant predictor for the proximal target (HR: 1.79, 95%CI: 1.05–3.05, *p* = 0.03). In our study, BED was a significant predictor of failure after GKS (OR: 0.996, 95%CI: 0.992–0.999, *p =* 0.02) for the entire cohort. An increase in BED reduced the risk of failure after GKS in the distal subgroup, with a threshold of 1850 Gy_2.47_ (OR: 19.44, 95%CI: 3.16–376.92, *p =* 0.01). In the case of proximal target, ID_50_ rather than BED, was the significant factor associated with failure (*p =* 0.02). Both our study and that of Warnick et al. (18) support the notion that a higher BED is beneficial for achieving initial pain relief in patients treated at the distal target. However, our study proposed a lower BED threshold. Notably, both studies employed the same BED model and radiobiological parameters, and the prescription doses for different targets were comparable. The divergence in findings may be attributed to differences in patient populations. Our cohort included patients with TN2 and MVC, whereas Warnick et al. (18) limited their analysis to TN1 and excluded cases involving vertebrobasilar artery compression. The multicenter nature, larger sample size, and greater consistency in patient selection in the study by Warnick et al. (18) likely contributed to more precise estimates of the BED effect, as reflected in the narrower confidence intervals. Consequently, it is possible that our study underestimated the optimal BED threshold for achieving initial pain relief at the distal target.

For long-term outcomes, BED was not a significant factor for the entire cohort or any of the targeting subgroups. Previous studies reported many factors influencing the long-term outcomes of TN patients after GKS, and the most believe that the appearance of complications means a lower risk of recurrence (8, 30, 32). In our study, the recurrence risk in patients with GKS-related complications was only 41% of that in others (HR: 0.41, 95%CI: 0.18–0.92, *p =* 0.03). We speculate that the presence of complications may mean more severe and complete radiational damage to the nerve, so patients with complications were at a lower recurrence risk. Additionally, our findings suggested that increased V_50_ (*p* = 0.002) and ID_50_ (*p* = 0.004) were significant predictors of a lower recurrence risk. Several studies have examined the role of nerve volume and ID in GKS, although results have been inconsistent. For instance, Barzaghi et al. (28) reported that an ID_50_ < 2.7 mJ predicted longer pain control (*p* = 0.043). Wolf et al. (33) reported that patients whose ratio of the integral dose over the total volume of the cisternal nerve was less than 0.05 were less likely to experience pain recurrence. Lovo et al. (34) reported that ID was associated with complications, but not with pain relief. The significant variability in these results may, in part, be attributed to differences in the measurement of nerve volume between centers. Contouring the target area often depends on the operator’s experience. Furthermore, the type of MRI imaging sequence and its resolution can affect the operator’s ability to accurately define the target boundary. For example, MRI cranial nerve water imaging can provide clearer morphological characteristics of the trigeminal nerve and often allows for automatic contouring through software. In contrast, T1-weighted MRI imaging relies on manual delineation by the operator. Variations in target delineation may lead to substantial discrepancies in the estimation of nerve volume and ID. However, BED is not influenced by these factors, which could explain the greater consistency in BED results reported across different centers.

In terms of the impact of BED on complications, we obtained consistent results with those of Tuleasca et al. (35). They found that the incidence of post-GKS hypoesthesia increased from <5% after a BED <1,800 Gy_2.47_ to 42% after a BED <2,600 Gy_2.47_. In our study, BED was a significant risk factor for post-GKS complications in the entire cohort and the distal subgroup. In distal targeting group, patients receiving BED >2,245 Gy_2.47_ had a risk of post-GKS complications more than three times of those receiving less BED (OR: 3.29, 95%CI: 1.23–9.44, *p =* 0.02). The latest multi-center study showed that physical dose, BED, and brainstem dose were not significant factors for the development of sensory dysfunction (18). However, when analyzed by target location, BED was correlated with the incidence of sensory dysfunction for distal targets (*p* = 0.02). Tuleasca et al. (35) noted a slight trend in the relationship between the incidence of hypoesthesia and the central dose, but this trend was not statistically significant. In our series, the central dose was also a significant risk factor for complications in the entire cohort and the distal subgroup. We explored all post-GKS complications, whereas they only investigated hypoesthesia, which may partly account for the differences in results. Across the entire cohort, the AUC for BED and complications was marginally lower than that for the physical dose (0.628 vs. 0.635), but for the distal target, BED had a higher AUC (0.690 vs. 0.664). This suggests that, for patients treated at the distal target, BED may be a better predictor of complications than the physical dose. But it must be admitted that the predictive strength of both BED and physical dose were rather limited. The effect of BED on pain relief and complications was observed only in the distal target subgroup. This difference may, in part, be attributed to the distinct radiobiological characteristics of the two irradiated target regions. Guclu et al. (20) provided anatomical evidence quantifying the central-to-peripheral myelin transition zone of the trigeminal nerve, reporting a mean length of 4.19 ± 0.81 mm from the brainstem, suggesting that this transition zone extends beyond the traditionally defined root entry zone (REZ). The proximal target is located near the transition zone, where the nerve myelin sheath undergoes a transition from oligodendrocytes to Schwann cells. This transitional area is believed to be particularly vulnerable to demyelination and ectopic excitation and is considered critical in the pathophysiology of trigeminal neuralgia. In contrast, the myelin sheath of the distal target is entirely composed of Schwann cells. Unlike oligodendrocytes—which are post-mitotic and exhibit limited regenerative capacity—Schwann cells retain proliferative potential and are capable of remyelinating injured axons, potentially conferring greater resistance to radiation exposure (36). These differences imply that the response to radiosurgery may vary depending on the myelin composition at the target site. Based on our results, for the distal target, increasing the BED enhanced initial pain relief but also raised the risk of complications. Therefore, there may be an optimal BED range that effectively balances complications and therapeutic efficacy. For the distal target, this range could be 1850–2,245 Gy_2.47_. Although current evidence does not definitively support a difference in overall efficacy between proximal and distal targets in GKS for TN, our findings suggest that BED optimization appears to confer greater therapeutic benefits at the distal target compared to the proximal target. When BED is properly adjusted, the distal target may offer a more favorable balance between pain relief and complications. Therefore, BED-guided dosing at the distal site may be a more advantageous strategy in GKS for TN. To validate this potential benefit, prospective studies are warranted. In particular, comparative studies involving different target locations receiving the same BED could more effectively elucidate the differential radiobiological responses of the trigeminal nerve subregions, thereby clarifying the specific impact of BED on treatment outcomes in TN.

There are several limitations in this study. Firstly, the retrospective and single-center nature of the study introduced selection bias and limited the generalizability of our findings. Secondly, the reliance on manual contouring by different neurosurgeon introduced potential inter-observer variability in target definition. Although all operators were experienced and followed a standardized protocol, subjective differences in interpretation, especially in challenging cases with unclear nerve margins, could have introduced measurement bias. Thirdly, unmeasured confounders such as medication adherence, psychological status, or differences in pain perception and reporting could have influenced the outcomes. These variables were not systematically captured in this study but are known to play a role in treatment response and patient satisfaction. Their absence may have introduced residual confounding, which could either attenuate or exaggerate the associations observed in this study. The last is related to the derivation of BED. Currently, all published studies of BED for GKS adopted the techniques described by Jones in 2019 or were based on their model with slight adjustments (24). Some radiobiological parameters used in the calculation of BED, including *c*, *k*, *μ*_1_, and *μ*_2,_ were obtained through animal experiments. Estimates based on these may inadequately reflect human trigeminal nerve responses due to fundamental biological differences. This is a critical source of systematic error. In addition, we noted that several published studies used the same radiobiological parameters in the BED calculation for different histological types, which is a helpless compromise due to the lack of specific parameters at the moment. Therefore, some of the results of this study should be interpreted with caution. More basic experiments are needed to obtain accurate radiobiological parameters so that the effect of BED on the clinical outcomes in TN patients who undergone GKS can be further verified.

## 5 Conclusion

GKS is an effective and safe treatment for primary TN. For TN patients treated at the distal target, BED is a significant predictor of both initial pain relief and post-GKS complications. But it is not a significant factor for long-term outcomes in either the entire cohort or any of the subgroups. Adjusting GKS doses according to BED for the distal target may optimize clinical outcomes in TN patients.

## Data Availability

The raw data supporting the conclusions of this article will be made available by the authors, without undue reservation.

## Footnotes

^1^ https://www.elekta.com/products/catalog/gamma-knife-radiosurgery/3-leksell-gamma-knife-perfexion/leksell-gammaplan/

^2^ https://cran.r-project.org/bin/windows/base/old/4.1.3/
