# Supplementary material for: Differential impact of biologically effective dose in distal versus proximal gamma knife targets for trigeminal neuralgia
Source: Front Neurol. 2025 Aug 25;16:1614981. doi: 10.3389/fneur.2025.1614981 (PMC12414993; doi:10.3389/fneur.2025.1614981)
Supplement: Supplementary file 1 [file Supplementary_file_1.docx]

Supplementary Material

# Supplementary Data

## Supplementary tables

| **TABLE S1. Univariate and multivariate logistic regression for failure in the distal subgroup** | | | | |
| --- | --- | --- | --- | --- |
|  | Univariate analysis | | Multivariate analysis | |
| Variables | OR (95%CI) | P | OR (95%CI) | P |
| Age | 1.00(0.93-1.08) | 0.95 |  |  |
| Sex | 0.51(0.11-2.34) | 0.37 |  |  |
| Duration between onset and GKS | 1.00(0.99-1.02) | 0.08 | 1.01(1.00-1.02) | 0.21 |
| Type of TN | 4.20(0.53-24.9) | 0.13 |  |  |
| BNI-PS before GKS | 3.25(0.7-15.22) | 0.12 |  |  |
| Previous surgery | 2.90(0.38-15.79) | 0.24 |  |  |
| Presence of MVC | 1.00(0.14-4.83) | 1.00 |  |  |
| Central dose | 0.70(0.46-1.00) | 0.06 |  |  |
| CDR | 0.16(0.01-0.85) | 0.08 |  |  |
| V_50_ | 0.78(0.3-1.72) | 0.58 |  |  |
| ID_50_ | 0.66(0.16-2.11) | 0.52 |  |  |
| BED | 0.996(0.992-0.999) | 0.02* | 0.996(0.993-0.999) | 0.03* |
| BED<1850Gy_2.47_ | 19.44(3.16-376.92) | 0.01* |  |  |

OR, odds ratio; CI, confident interval; GKS, Gamma knife radiosurgery; TN, trigeminal neuralgia; BNI-PS, Barrow Neurological Institute Pain Scale; MVC, microvascular decompression; CDR, dose rate of Co^60^; V_50_, nerve volume inside the 50% isodose line; ID_50_, integral dose inside the 50% isodose line; BED, biologically effective dose. **p*<0.05

| **TABLE S2. Univariate and multivariate COX regression for recurrence in overall group** | | | | |
| --- | --- | --- | --- | --- |
|  | Univariate analysis | | Multivariate analysis | |
| Variables | HR (95%CI) | P | HR (95%CI) | P |
| Age | 0.99(0.96-1.03) | 0.77 |  |  |
| Sex | 0.57(0.26-1.23) | 0.15 |  |  |
| Duration between onset and GKS | 1.00(0.99-1.01) | 0.23 |  |  |
| Type of TN | 2.10(0.72-6.13) | 0.17 |  |  |
| BNI-PS before GKS | 0.40(0.12-1.35) | 0.14 |  |  |
| Previous surgery | 1.13(0.39-3.27) | 0.82 |  |  |
| Presence of MVC | 1.63(0.73-3.61) | 0.23 |  |  |
| Central dose | 0.99(0.91-1.07) | 0.81 |  |  |
| CDR | 1.06(0.56-2.02) | 0.86 |  |  |
| V_50_ | 0.44(0.26-0.75) | 0.002* |  |  |
| ID_50_ | 0.32(0.15-0.71) | 0.004* | 0.34(0.15-0.78) | 0.01* |
| ID_50_>1.9mJ | 0.37(0.14-0.99) | 0.04* |  |  |
| BED | 1.00(0.999-1.002) | 0.58 |  |  |
| BED>1853Gy_2.47_ | 1.80(0.71-4.57) | 0.22 |  |  |
| Target | 2.13(0.94-4.84) | 0.07 | 1.82(0.77-4.27) | 0.17 |
| Presence of complications | 0.43(0.19-0.96) | 0.04* | 0.41(0.18-0.92) | 0.03* |

HR, hazard ratio; CI, confident interval; GKS, Gamma knife radiosurgery; TN, trigeminal neuralgia; BNI-PS, Barrow Neurological Institute Pain Scale; MVC, microvascular decompression; CDR, dose rate of Co^60^; V_50_, nerve volume inside the 50% isodose line; ID_50_, integral dose inside the 50% isodose line; BED, biologically effective dose.

**p*<0.05

| **TABLE S3. Univariate and multivariate COX regression for recurrence in the proximal subgroup** | | | | |
| --- | --- | --- | --- | --- |
|  | Univariate analysis | | Multivariate analysis | |
| Variables | HR (95%CI) | P | HR (95%CI) | P |
| Age | 0.98(0.92-1.04) | 0.49 |  |  |
| Sex | 0.39(0.12-1.29) | 0.13 |  |  |
| Duration between onset and GKS | 0.99(0.98-1.00) | 0.17 |  |  |
| Type of TN | 4.54(1.20-17.18) | 0.03* | 3.6(0.94-13.82) | 0.06 |
| BNI-PS before GKS | 1.02(0.23-4.73) | 0.97 |  |  |
| Previous surgery | 2.13(0.62-7.30) | 0.23 |  |  |
| Presence of MVC | 1.63(0.52-5.05) | 0.40 |  |  |
| Central dose | 1.02(0.82-1.27) | 0.84 |  |  |
| CDR | 0.68(0.23-2.04) | 0.50 |  |  |
| V_50_ | 0.70(0.29-1.73) | 0.44 |  |  |
| ID_50_ | 0.64(0.17-2.39) | 0.51 |  |  |
| BED | 1.001(0.999-1.003) | 0.55 |  |  |
| BED>2237Gy_2.47_ | 1.63(0.42-6.25) | 0.48 |  |  |
| Presence of complications | 0.25(0.06-0.93) | 0.04* | 0.28(0.07-1.07) | 0.06 |

HR, hazard ratio; CI, confident interval; GKS, Gamma knife radiosurgery; TN, trigeminal neuralgia; BNI-PS, Barrow Neurological Institute Pain Scale; MVC, microvascular decompression; CDR, dose rate of Co^60^; V_50_, nerve volume inside the 50% isodose line; ID_50_, integral dose inside the 50% isodose line; BED, biologically effective dose.

**p*<0.05

| **TABLE S4. Univariate and multivariate logistic regression for post-GKS complication in overall group** | | | | |
| --- | --- | --- | --- | --- |
|  | Univariate analysis | | Multivariate analysis | |
| Variables | OR (95%CI) | P | OR (95%CI) | P |
| Age | 1.00(0.96-1.03) | 0.83 |  |  |
| Sex | 2.05(1.00-4.26) | 0.05 | 1.76(0.83-3.80) | 0.14 |
| Duration between onset and GKS | 1.00(0.99-1.01) | 0.10 | 1.005(1-1.011) | 0.06 |
| Type of TN | 0.35(0.08-1.20) | 0.12 |  |  |
| BNI-PS before GKS | 2.09(0.92-4.92) | 0.08 | 1.81(0.75-4.46) | 0.19 |
| Previous surgery | 0.64(0.81-1.94) | 0.64 |  |  |
| Presence of MVC | 0.80(0.38-1.67) | 0.56 |  |  |
| Central dose | 1.10(1.03-1.17) | 0.01* |  |  |
| Central dose>84.5 Gy | 3.21(1.61-6.59) | 0.001* |  |  |
| CDR | 1.12(0.64-1.95) | 0.69 |  |  |
| CDR>1.886 Gy/min | 1.58(0.80-3.15) | 0.19 |  |  |
| V_50_ | 0.89(0.59-1.33) | 0.57 |  |  |
| ID_50_ | 1.05(0.58-1.88) | 0.88 |  |  |
| BED | 1.002(1.000-1.003) | 0.01* | 1.002(1-1.003) | 0.01* |
| BED>2086Gy_2.47_ | 2.94(1.45-6.05) | 0.003* |  |  |
| Target | 0.67(0.33-1.31) | 0.24 |  |  |

OR, odds ratio; CI, confident interval; GKS, Gamma knife radiosurgery; TN, trigeminal neuralgia; BNI-PS, Barrow Neurological Institute Pain Scale; MVC, microvascular decompression; CDR, dose rate of Co^60^; V_50_, nerve volume inside the 50% isodose line; ID_50_, integral dose inside the 50% isodose line; BED, biologically effective dose.

**p*<0.05

| **TABLE S5. Univariate and multivariate logistic regression for post-GKS complication in the distal target** | | | | |
| --- | --- | --- | --- | --- |
|  | Univariate analysis | | Multivariate analysis | |
| Variables | OR (95%CI) | P | OR (95%CI) | P |
| Age | 1.00(0.96-1.05) | 0.96 |  |  |
| Sex | 3.44(1.3-9.81) | 0.02* | 2.97(1.07-8.73) | 0.04* |
| Duration between onset and GKS | 1.00(1.00-1.01) | 0.36 |  |  |
| Type of TN | 0.39(0.05-1.94) | 0.28 |  |  |
| BNI-PS before GKS | 1.86(0.67-5.42) | 0.24 |  |  |
| Previous surgery | 1.37(0.33-5.95) | 0.66 |  |  |
| Presence of MVC | 0.93(0.32-2.65) | 0.89 |  |  |
| Central dose | 1.40(1.10-1.83) | 0.01* |  |  |
| Central dose>82 Gy | 5.16(1.64-19.88) | 0.01* |  |  |
| CDR | 1.95(0.90-4.39) | 0.10 |  |  |
| V_50_ | 0.87(0.52-1.42) | 0.57 |  |  |
| ID_50_ | 0.99(0.48-2.03) | 0.97 |  |  |
| BED | 1.002(1.000-1.004) | 0.01* | 1.000(1.002-1.004) | 0.02* |
| BED>2245Gy_2.47_ | 3.29(1.23-9.44) | 0.02* |  |  |

OR, odds ratio; CI, confident interval; GKS, Gamma knife radiosurgery; TN, trigeminal neuralgia; BNI-PS, Barrow Neurological Institute Pain Scale; MVC, microvascular decompression; CDR, dose rate of Co^60^; V_50_, nerve volume inside the 50% isodose line; ID_50_, integral dose inside the 50% isodose line; BED, biologically effective dose.

**p*<0.05

## Supplementary Figure


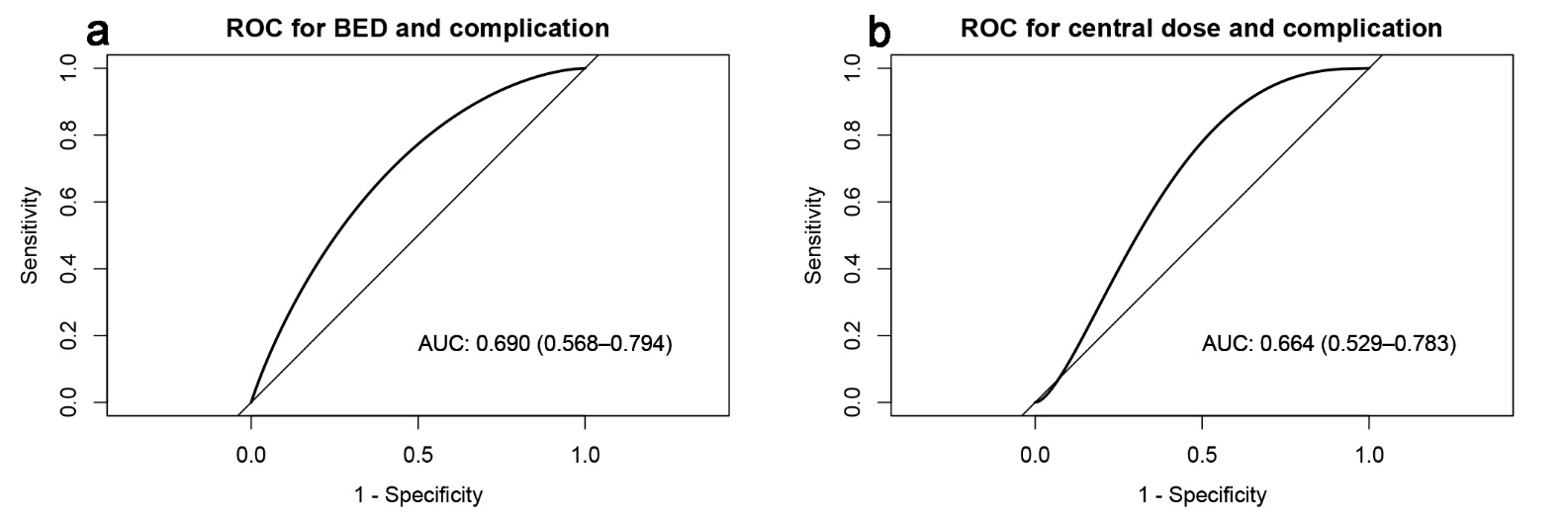


**Supplementary Figure. 2 (a)** ROC curve for BED and complication in the distal subgroup. **(b)** ROC curve for physical dose and complication in the distal subgroup.

# Supplementary Method

We adopted the model described by Jones et al. in 2019 to calculate BED (1). An *α/β* ratio of 2.47Gy was used. The model based on biexponential DNA repair kinetics compensated for the impact of treatment duration on radiation biological effects. Since only patients with single exposure were included in this study, we simplified the equation as follow:

$$\boldsymbol{BED}\boldsymbol{=}\frac{\boldsymbol{d}}{\boldsymbol{1+c}}\left[ \boldsymbol{1+}\frac{\boldsymbol{d}}{\boldsymbol{k}}\boldsymbol{f}\left( \boldsymbol{\mu}_{\boldsymbol{1}}\boldsymbol{t} \right) \right]\boldsymbol{+}\frac{\boldsymbol{cd}}{\boldsymbol{1+c}}\left[ \boldsymbol{1+}\frac{\boldsymbol{d}}{\boldsymbol{k}}\boldsymbol{f}\left( \boldsymbol{\mu}_{\boldsymbol{2}}\boldsymbol{t} \right) \right]$$

where *c* is the partition coefficient associated with the fast/slow two-phase repair rate of sublethal radiation damage. *k=α/β* is a tissue-specific constant. The *α/β* ratio refers to the single dose at which the lethal and sublethal injury of irradiation have the same cytotoxic effect. The parameter *µ* refers to sublethal radiation damage repair rates under protracted irradiation. *µ_1_* represents the fast phase repair rate, and *µ_2_* is the slow rate. This study used the repair kinetic parameters of the white matter of the spinal cord in rats measured by Pop et al(2). With a partition coefficient (*c*) of 0.98, *µ_1_* and *µ_2_* are 3.65 and 0.32 per hour, respectively, according to Pop et al. *t* is the average time spent on each shot. *f(µt)* is a function associated with sublethal damage repair and time(1). The function is as follows:

$$\boldsymbol{f}\left( \boldsymbol{\mu t} \right)\boldsymbol{=}\frac{\boldsymbol{2}}{\boldsymbol{\mu t}}\left[ \boldsymbol{1-}\frac{\left( \boldsymbol{1-}\boldsymbol{e}^{\boldsymbol{-\mu t}} \right)}{\boldsymbol{1-\mu t}} \right]$$

Reference:

1. Jones B, Hopewell JW. Modelling the influence of treatment time on the biological effectiveness of single radiosurgery treatments: derivation of "protective" dose modification factors. Br J Radiol. 2019;92(1093):20180111.

2. Pop LA, Millar WT, van der Plas M, van der Kogel AJ. Radiation tolerance of rat spinal cord to pulsed dose rate (PDR-) brachytherapy: the impact of differences in temporal dose distribution. Radiother Oncol. 2000;55(3):301-15.
